# Systemic and pulmonary C1q as biomarker of progressive disease in experimental non-human primate tuberculosis

**DOI:** 10.1038/s41598-020-63041-4

**Published:** 2020-04-14

**Authors:** Karin Dijkman, Rosalie Lubbers, Nicole V. Borggreven, Tom H. M. Ottenhoff, Simone A. Joosten, Leendert A. Trouw, Frank A. W. Verreck

**Affiliations:** 1 Section of TB Research & Immunology, department of Parasitology, Biomedical Primate Research Centre (BPRC), Rijswijk, the Netherlands; 2 The department of Rheumatology, Leiden University Medical Centre (LUMC), Leiden, the Netherlands; 3 The department of Immunohematology and Blood Transfusion, Leiden University Medical Centre (LUMC), Leiden, the Netherlands; 4 The department of Infectious Diseases, Leiden University Medical Centre (LUMC), Leiden, the Netherlands

**Keywords:** Complement cascade, Tuberculosis, Translational immunology, Diagnostic markers, Experimental models of disease

## Abstract

Tuberculosis (TB) causes 1.6 million deaths annually. Early differential diagnosis of active TB infection is essential in optimizing treatment and reducing TB mortality, but is hampered by a lack of accurate and accessible diagnostics. Previously, we reported on complement component C1q, measured in serum by ELISA, as a candidate biomarker for active tuberculosis. In this work we further examine the dynamics of C1q as a marker of progressive TB disease in non-human primates (NHP). We assessed systemic and pulmonary C1q levels after experimental infection using high or low single dose as well as repeated limiting dose *Mycobacterium tuberculosis* (*Mtb*) challenge of macaques. We show that increasing C1q levels, either peripherally or locally, correlate with progressive TB disease, assessed by PET-CT imaging or post-mortem evaluation. Upregulation of C1q did not precede detection of *Mtb* infection by a conventional interferon-gamma release assay, confirming its association with disease progression. Finally, pulmonary vaccination with Bacillus Calmette Guérin also increased local production of C1q, which might contribute to the generation of pulmonary protective immunity. Our data demonstrate that NHP modelling of TB can be utilized to study the role of C1q as a liquid biomarker in TB protection and disease, complementing findings in TB patients.

## Introduction

Tuberculosis (TB) remains a highly significant burden to global health. In 2018, 6.4 million new cases of TB were officially notified to the World Health Organization (WHO)^1^. However, the WHO estimates the actual number of TB cases to be 10 million^1^, implying an underestimation of the true number of TB cases. Closing this gap in TB detection has the potential to prevent millions of deaths and to curb further dissemination of TB. Accordingly, the WHO has made early diagnosis of tuberculosis an integral part of their EndTB strategy.

After infection with *Mycobacterium tuberculosis* (*Mtb*), the causative agent of TB, individuals may develop latent TB infection (LTBI) or active progressive disease (although some may also clear the infection). Definitive diagnosis of active TB can only be made by detection of *Mycobacterium tuberculosis* (*Mtb*) in sputum, either by smear microscopy, culture or GeneXpert technology. These assays, however, are time-consuming, require specific (expensive) infrastructure and suffer from sampling error and low sensitivity. Immunological tests, such as Tuberculin Skin Testing (TST) or Interferon Gamma Release Assays (IGRAs) can detect exposure to mycobacteria, but are unable to differentiate between active and latent TB infection^2,3^. These host response measures can remain positive even after treatment and clearance of infection. Accurate tests for risk of relapse after drug-treatment or risk of progression to active disease are currently unavailable. A wide range of new TB diagnostics are currently under development, investigating the diagnostic potential of pathogen-derived components as well as host-derived biomarkers in various body fluids^2,3^.

Over the last few years many host-derived candidate biomarkers of progressive TB disease have been identified, and amongst them several components of the complement system^4–9^. The complement system consists of a number of serum proteins with the capacity to recognize and neutralize invading pathogens by opsonization and lysis. One of these components, C1q, can bind to pathogen-bound C-reactive protein, IgG or IgM, thereby activating the complement cascade through its associated serine proteases C1s and C1r. This binding results in subsequent deposition of the opsonin C3b on the surface of the pathogen and, ultimately, the formation of the membrane attack complex (MAC). Additionally, C1q enhances phagocytosis and has been implied, amongst others, in tissue repair, synaptic pruning and macrophage polarization (reviewed in^10,11^). More recently, a role for complement components in shaping innate and adaptive immune responses has been established. For instance, in a model for systemic lupus erythematosus, C1q was found to control auto-reactive CD8+ T-cell responses, by modulating the mitochondrial metabolism of these T-cells^12,13^. C1q is mainly produced by cells of the myeloid lineage^14^, and especially by immature dendritic cells^15^, macrophages^16^ and mast cells^17^.

We and others have previously described serum C1q as a biomarker of active TB disease^18,19^. In patients with active TB we observed significantly higher levels of serum C1q compared to individuals with latent *Mtb* infection or individuals with non-mycobacterial pneumonia. Furthermore, we showed increase of C1q after high dose TB challenge of Non-Human Primates (NHPs).

NHP and macaque species (*Macaca spp*) in particular, are considered highly relevant models for TB, due to their close phylogenetic relationship to man, outbred nature and large similarity in TB pathogenesis. Macaques are applied across the whole spectrum of TB research, both in preclinical evaluation of TB vaccines and therapeutics as well as basic research on TB disease development^20–22^. Modelling in these species presents the advantage of having controlled and accurate time-response-conditions relative to infection. Depending on macaque (sub)species, *M.tuberculosis* strain and challenge dose, TB disease manifestation in macaques mimics the diversity seen in humans^23–25^.

In this work, we exploited the diversity in TB disease manifestation in NHPs to examine the dynamics of C1q as a biomarker of TB disease in more detail. We assessed C1q levels in plasma and bronchoalveolar lavages (BALs) in multiple independent cohorts of *Mtb* infected macaques under varying experimental challenge conditions. By profiling circulating and pulmonary C1q levels at various timepoints after infection we show that increasing C1q levels correlate with increased TB pathology and with decreased survival following challenge with high or low dose *Mtb*. However, neither peripheral nor local upregulation of C1q preceded IGRA conversion, suggesting its association with progressive disease but not TB infection per se. Lastly, we show that pulmonary vaccination with Bacillus Calmette Guérin (BCG), which is superior in inducing protection against TB compared to standard intradermal vaccination^23,26,27^, also results in an increase in pulmonary C1q and discuss its potential role in the generation of a protective immune responses. Our observations confirm and further support C1q as a marker of progressive TB disease.

## Results

### C1q is a predictive marker of disease progression in NHP TB

In addition to having described serum C1q as a biomarker of active TB in human patients, we also reported on elevated C1q levels in a preliminary analysis of serum and BAL samples from *Mtb*-infected non-human primates^18^. Here we set out to further investigate C1q as a marker of TB disease, under various model conditions in NHPs.

**Figure 1:**
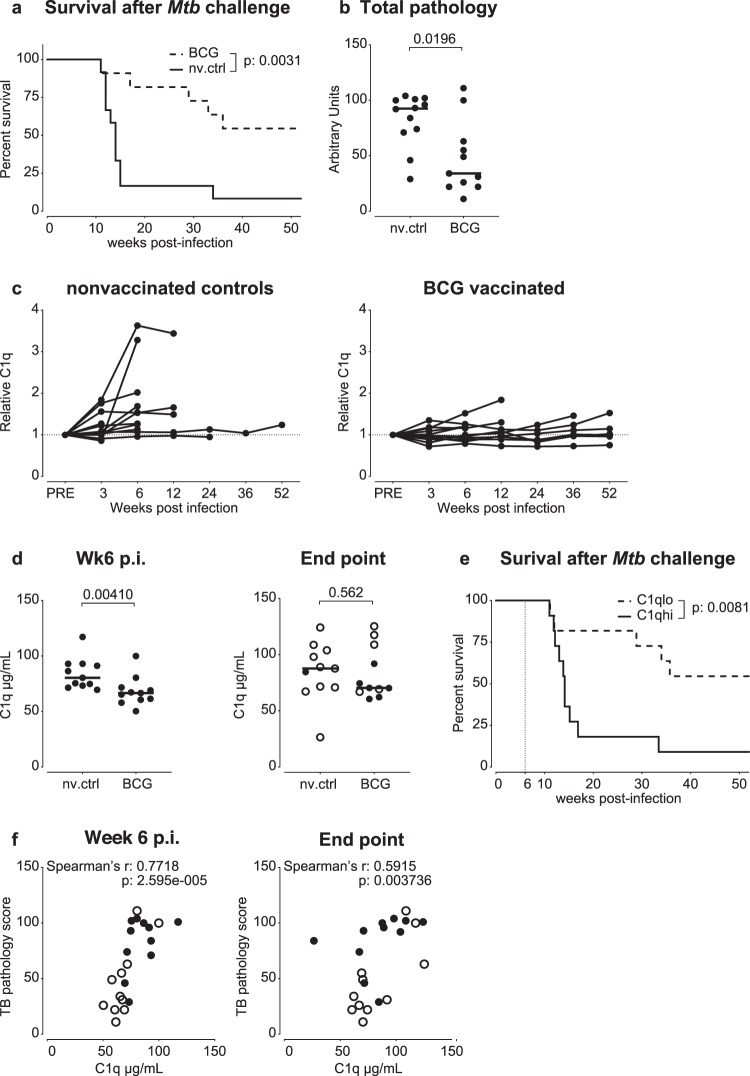
C1q is a predictive marker of disease progression in NHP TB. Increase in serum C1q correlates with disease manifestation after high dose *Mtb* challenge. (**a**) Kaplan-Meier curves of time-to-endpoint (survival) for non-vaccinated (solid line, n = 12) and BCG vaccinated (dashed line, n = 11) rhesus macaques after infection with 500 CFU of *Mtb*. (**b**) Total post-mortem tuberculosis pathology scores, showing reduction of TB disease by prior BCG vaccination. (**c**) Serum C1q levels relative to pre-infection values of nonvaccinated controls and BCG vaccinees. (**d**) Group-comparison of serum C1q concentration 6 weeks post-infection (p.i.) and at endpoint. Open symbols in right panel represent animals that reached a humane endpoint. (**e**) Kaplan-Meier curves of time-to-endpoint (survival) for animals with high/above median (solid line) or low/below median C1q levels (dashed line) at 6 weeks post-infection. (**f**) Correlation of serum C1q levels at 6 weeks post-infection and at endpoint, with total tuberculosis pathology scores from BCG vaccinated animals represented by open symbols. Horizontal lines in (**b**) & (**d**) indicate group medians. Statistical significance of group differences determined by two sided Mann-Whitney. Statistical curve comparison in (**a**) and (**e**) by Mantel-Cox Log-rank test. Correlations in (**f**) calculated with Spearman’s rank-order test.

We started with retrospective measurement of C1q in serum from rhesus macaques (*Macaca mulatta*) that were either vaccinated or not with a standard human dose of intradermal BCG and subsequently challenged with a high dose (500 Colony Forming Units (CFU)) of *Mtb* strain Erdman, previously described in part in Lubbers *et al*.^18^. These animals were monitored for 1 year after infectious challenge, or until reaching a humane endpoint due to progressive TB disease. In this cohort, prior BCG vaccination was efficacious and resulted in significant improvement of survival after *Mtb* challenge (p = 0.0031, Fig. 1a) and reduction of TB associated pathology (p = 0.0196, Fig. 1b).

Serum C1q levels did not differ between the two groups prior to infection (Supplemental Fig. 1a). Assessing changes over time, we observed most prominent upregulation in non-vaccinated controls (nv.ctrls) already from 3 weeks after infection with *Mtb* (Fig. 1c). In some individuals, an increase in C1q levels preceded a humane endpoint event by several weeks. By week 6 post-*Mtb* infection, group median serum C1q in non-vaccinated controls was significantly higher compared to BCG vaccinees (Fig. 1d, left panel). Despite reducing TB pathology by prior BCG vaccination (Fig. 1b), when comparing C1q levels at individuals’ endpoints, non-vaccinated controls and BCG vaccinated animals showed no significant difference in serum C1q (Fig. 1d, right panel).

To determine the potential of serum C1q as a prognostic marker regardless of prophylactic treatment and prior to the incidence of humane endpoints, we divided all animals in two groups irrespective of treatment: those exhibiting week 6 C1q levels below the median value (C1q.lo, 2 nv.ctrl and 9 BCG) and animals with week 6 levels above the median (C1q.hi, 9 nv.ctrl and 2 BCG). When comparing Kaplan-Meier curves for time-to-endpoint, animals with the highest C1q levels displayed significantly reduced survival (p = 0.0081, Fig. 1e). Furthermore, we found a strong statistical correlation between the total TB pathology score at necropsy and serum C1q levels at 6 weeks post-infection (Spearman’s rho = 0.772, p < 0.0001, Fig. 1f, left panel). Individual C1q levels at endpoint correlated significantly with the total amount of pathology (Spearman’s rho = 0.591, p = 0.003, Fig. 1f, right panel). Taken together, these observations suggest that C1q is an early marker of progressive disease after experimental infection of rhesus macaques.

### C1q is a marker of differential disease severity in distinctive rhesus cohorts

**Figure 2:**
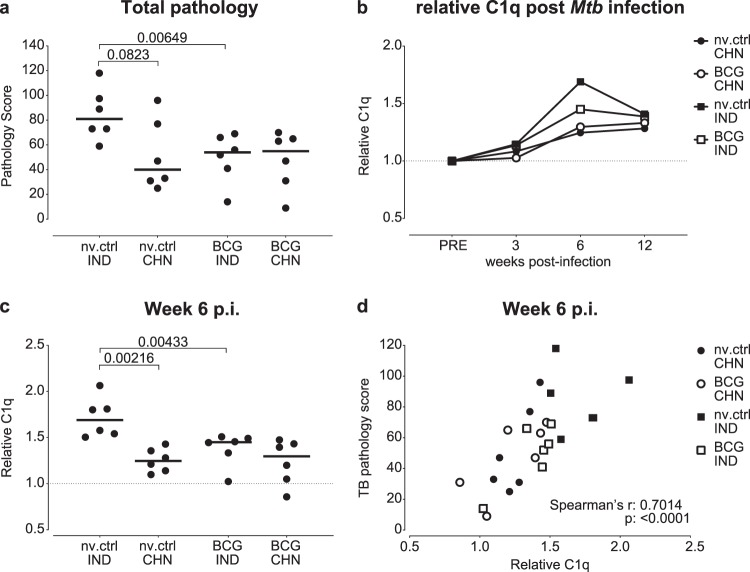
C1q is a marker of differential disease severity in distinctive rhesus cohorts. Serum C1q levels after *Mtb* infection of two genotypic cohorts of rhesus macaques. (**a**) Total tuberculosis pathology scores after infection of unvaccinated (nv.ctrl) or BCG vaccinated rhesus macaques with 500 CFU of *Mtb*. IND: Indian genotype, CHN: Chinese genotype. N = 6 per group. (**b**) Group medians of relative C1q levels over the course of *Mtb* infection. (**c**) Group-comparison of relative C1q levels at 6 weeks post-infection (p.i). (**d**) Correlation of relative C1q levels with total tuberculosis pathology scores. Horizontal lines in (**a**) and (**c**) indicate group medians. Statistical significance of group differences determined by two sided Mann-Whitney. Correlation in (**d**) calculated with Spearman’s rank-order test.

We next sought to confirm the correlation of C1q upregulation with disease severity in an independent cohort of *Mtb* infected NHP. As a species, rhesus macaques can be divided into distinct subpopulations based on genetic variation that is associated with their geographical distribution^28^. When comparing Chinese versus Indian type rhesus macaques head-to-head, we found the latter to exhibit a more severe TB disease phenotype, 12 weeks after single high dose (500 CFU) *Mtb* challenge (^23^ & Fig. 2a, non-vaccinated controls). Prior intradermal BCG vaccination reduced TB associated pathology in Indian but not Chinese type rhesus macaques. We measured C1q in serum collected at 3 weekly intervals and assessed the association of C1q levels with TB pathology.

As observed previously, serum C1q levels increased during experimental infection with *Mtb*, from 3 weeks post-infection onward (Fig. 2b). The strongest increase in serum C1q was again observed in the animals with the highest disease severity, the non-vaccinated Indian rhesus macaques (Fig. 2b). Reduced TB pathology, as seen in Chinese type rhesus macaques or after prior BCG vaccination of Indian type rhesus macaques, is reflected in the limited upregulation of C1q in these groups (Fig. 2b). Since we observed a trend of higher C1q levels prior to infection in BCG vaccinated Chinese rhesus macaques in particular (Supplemental Fig. 1b), we used individual fold-increase of C1q (a relative measure of C1q) in subsequent analyses. When comparing values 6 weeks post-infection, relative C1q values were significantly higher in unvaccinated Indian rhesus macaques compared to BCG vaccinated Indian rhesus macaques or unvaccinated Chinese type macaques (Fig. 2c). Non-parametric Spearman’s analysis of the relative C1q levels at week 6 versus total TB pathology scores revealed a strong correlation (rho = 0.7014, p = <0.0001, Fig. 2d), corroborating the association between C1q upregulation and tuberculosis disease severity.

### Increased C1q is observed in the pulmonary space after *Mtb* infection

**Figure 3:**
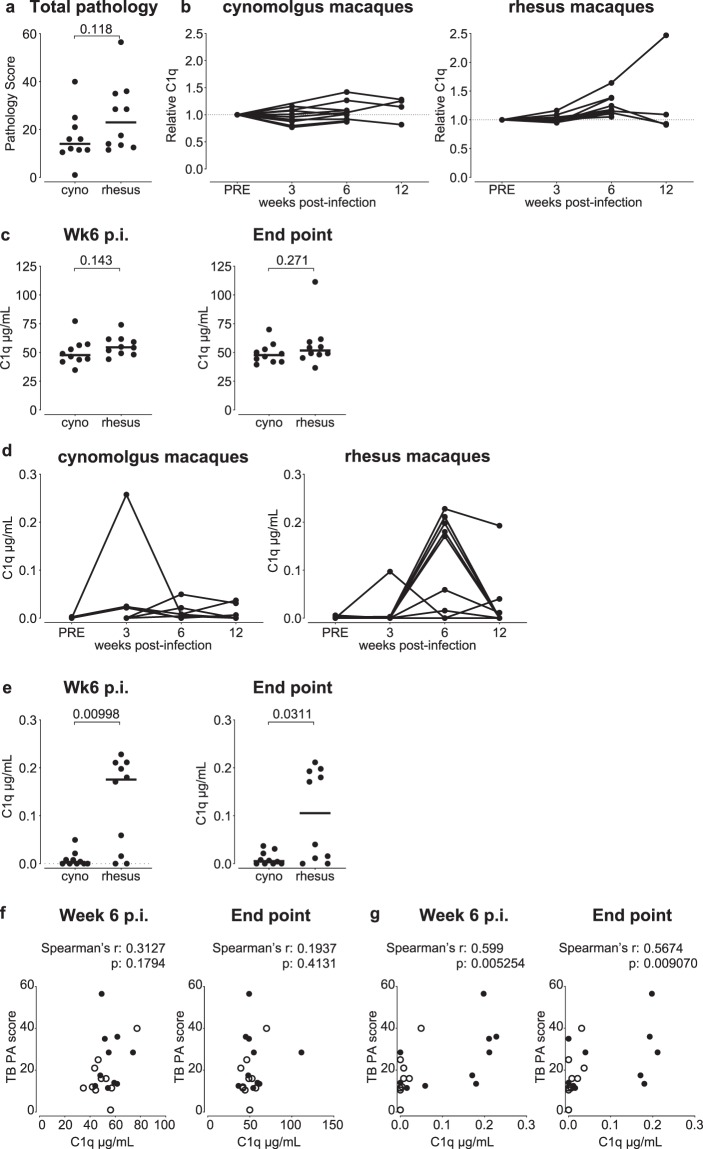
Increase in local C1q in TB disease susceptible rhesus but not cynomolgus macaques. Analysis of C1q levels in serum and broncho-alveolar lavage (BAL) fluid after low dose (1–7 CFU) *Mtb* challenge of rhesus and cynomolgus macaques (n = 10 per species). (**a**) Total tuberculosis pathology scores after *Mtb* infection of rhesus versus cynomolgus macaques. (**b**) Relative levels of serum C1q over the course of *Mtb* infection for the two species. (**c**) Species comparison of serum C1q concentration 6 weeks post-infection (p.i, left panel) and at endpoint, being either week 6 or week 12 post-infection by predefined study plan (right panel). (**d**) BAL C1q levels over the course of *Mtb* infection for both species. (**e**) Species comparison of BAL C1q levels 6 weeks post-infection (p.i) and at endpoint. Correlation of (**f**) serum C1q and (**g**) BAL C1q levels, both at 6 weeks post-infection and at endpoint, with total amount of tuberculosis pathology. Horizontal lines in (**a**), (**c**) and (**e**) indicate group medians. Open symbols in (**f**) and (**g**) represent cynomolgus macaques; closed symbols rhesus macaques. Statistical significance of group differences determined by two sided Mann-Whitney. Correlations in (**f**) and (**g**) calculated with Spearman’s rank-order test.

Over time we have moved away from high dose infection studies, since macaques appeared highly susceptible to infection and natural exposure to *Mtb* likely occurs at much lower doses than the 500 CFU applied in the studies described above^29^. To investigate whether increasing serum C1q still associates with disease severity after low dose *Mtb* infection, we assessed serum C1q dynamics in a cohort consisting of rhesus and cynomolgus macaques (*Macaca mulatta* and *Macaca fascicularis*, respectively) that were infected with a low dose (<10 CFU) of *Mtb*. The two species differ in the extent of TB pathology development after experimental infection; rhesus macaques typically tend to develop more severe TB pathology (Fig. 3a,^30^,^31^). These animals were sacrificed either 6 or 12 weeks after *Mtb* infection, and displayed significantly less TB pathology compared to the animals from the studies described in Figs. 1 and 2 (Supplemental Fig. 2).

Baseline pre-infection levels of serum C1q did not differ between the two species (Supplemental Fig. 1c). After infection with low dose *Mtb*, overall we observed a modest increase in serum C1q only, most apparent between 3 and 6 weeks post-infection (with the exception of one rhesus showing a progressive increase) (Fig. 3b). However, despite increased TB disease levels in rhesus over cynomolgus macaques, there was no significant difference in C1q levels between the two species, neither when comparing C1q levels at week 6 post-infection nor at endpoint (that is pooling endpoint values of animals sacrificed at week 6 or 12) (Fig. 3c, left and right panel, respectively).

Considering the relatively low serum C1q levels in these animals after low dose *Mtb* challenge, we interrogated C1q at the site of infection by measuring C1q in BAL fluids collected at various timepoints post-infection. Prior to *Mtb* infection, C1q was virtually undetectable in BAL fluids from either species (Supplemental Fig. 1c, right panel). However, from 3 to 6 weeks post-infection we observed a marked increase in C1q in the BALs of rhesus macaques (Fig. 3d), though C1q levels dropped between week 6 and week 12. In contrast, this marked increase in local C1q was absent in cynomolgus macaques (except for one animal with an (unexplained) outlier measurement at week 3). When comparing C1q values at 6 weeks post-infection and at study endpoint, increased C1q levels were observed in the rhesus but not the cynomolgus cohort (Fig. 3e). As expected from the above findings, serum C1q levels did not correlate with TB disease scores (Fig. 3f). But, when analyzing C1q expression in BAL 6 weeks post-challenge, we did find a statistically significant correlation with TB pathology (Spearman’s rho = 0.599, p = 0.005, Fig. 3g). Thus, also after low dose *Mtb* challenge and subsequent mild(er) TB disease, increasing C1q levels remain associated with disease progression and pathological involvement, albeit locally rather than peripherally.

### C1q upregulation does not precede IGRA conversion

**Figure 4:**
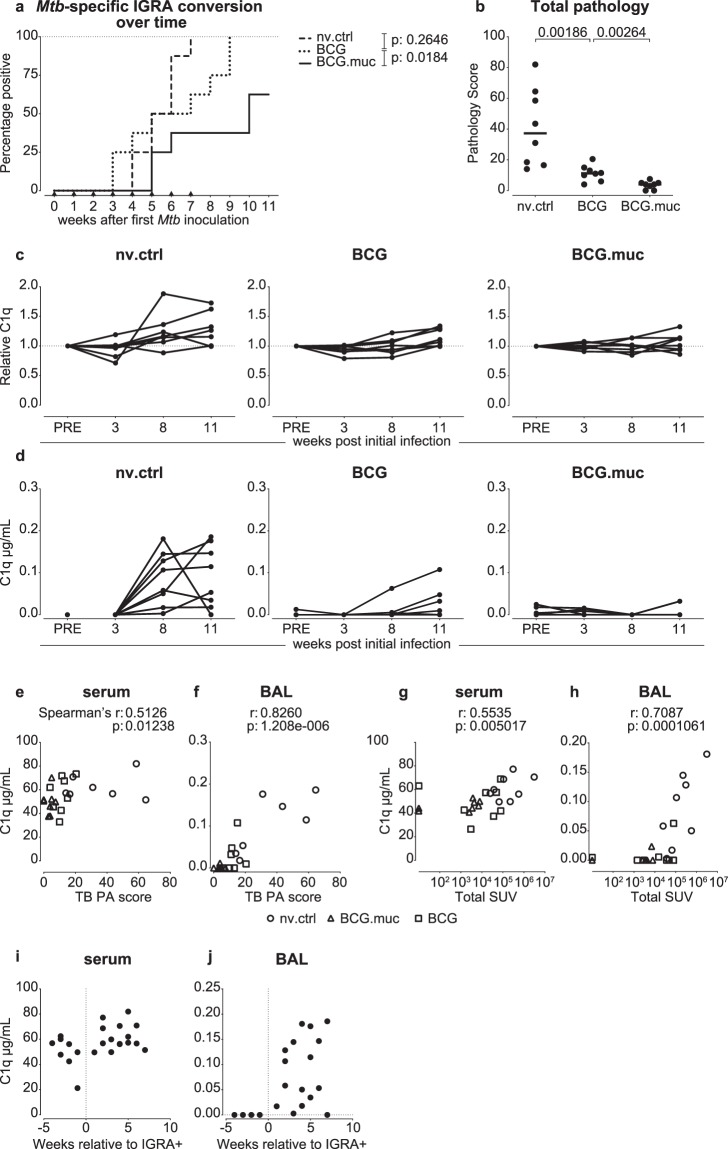
Infection-associated IGRA conversion precedes peripheral and local C1q increase. Analysis of C1q levels in serum and broncho-alveolar lavage (BAL) fluid after repeated limiting dose (RLD) *Mtb* challenge of rhesus macaques that were either vaccinated by standard intradermal (BCG) or pulmonary mucosal (BCG.muc) BCG administration or left untreated (nv.ctrl). (**a**) Rate of IGRA conversion after RLD *Mtb* challenge in vaccinated and non-vaccinated animals. (**b**) Total tuberculosis pathology scores per treatment group after RLD *Mtb* challenge. C1q levels over the course of *Mtb* infection in (**c**) serum and (**d**) BAL fluid. Correlation between (**e**) serum C1q and (**f**) BAL C1q levels 11 weeks after the first exposure to *Mtb* and the total amount of tuberculosis pathology. Correlation between (**g**) serum C1q and (**h**) BAL C1q levels and total lung standard uptake values (SUV) as measured by PET-CT 8 weeks after initial Mtb challenge. (**i**) Serum and (**j**) BAL C1q levels of non-vaccinated control animals over time, aligned relative to IGRA conversion. Horizontal lines in (**b**) indicate group medians. Statistical significance of group differences determined by two sided Mann-Whitney. Correlations in (**e–h**) calculated with Spearman’s rank-order test.

Having established that C1q is upregulated either peripherally and/or locally in association with various TB (challenge and) disease levels, we subsequently wanted to investigate if this increase in C1q could serve as a diagnostic marker of *Mtb* infection, with superior sensitivity to a conventional IFNγ Release Assay (IGRA). To address the diagnostic potential of C1q measurement, we profiled peripheral and local C1q levels in a challenge study in rhesus macaques in which we deployed a novel repeated limiting dose (RLD) *Mtb* challenge modality. In this RLD challenge model, we have demonstrated prevention of infection and disease after mucosal BCG vaccination (Fig. 4a,b, previously published and further detailed in Dijkman *et al*.^26^, and depicted here only to provide relevant background).

As observed after single low dose infection, RLD *Mtb* challenge resulted in a modest increase in serum C1q over the course of the infection phase, in particular in non-vaccinated control animals, which, expectedly displayed the highest tuberculosis pathology levels at study end-point (Fig. 4c). Animals protected from severe TB disease by prior BCG vaccination (by either standard intradermal or pulmonary mucosal route of administration) did not show any increase in relative serum C1q. Similarly, C1q is markedly increased in BAL fluids of the non-vaccinated control group, but not after prior BCG vaccination (with the exception of a few intradermally BCG vaccinated animals) (Fig. 4d). Also in this study, we found BAL as well as serum C1q levels to statistically correlate with the level of TB pathology at endpoint, once more confirming C1q upregulation to be associated with disease development and severity rather than infection (Fig. 4e,f). Along *Mtb* infection (rather than post mortem), disease severity can be assessed by means of PET-CT imaging, in which inflammation and metabolically active granulomas can be visualized using ^18^F-fluorodeoxyglucose (FDG) as a PET tracer^32^. To investigate whether C1q levels correlate with TB pathology as measured by PET-CT, we plotted summed lung and bronchoalveolar Standard Uptake Values (SUVs) obtained 8 weeks after initial challenge against C1q levels measured in serum and BAL collected at that same timepoint. Again, we found both serum and BAL fluid C1q concentrations to correlate significantly (p < 0.05) with PET-CT signals of TB pathology (Spearman’s rho 0.554 and 0.709 respectively, Fig. 4g,h).

We next set out to investigate if C1q upregulation preceded detection of *Mtb* infection by IGRA conversion and therefore aligned the C1q responses in the non-vaccinated control animals to their time point of IGRA conversion. As can be expected for a marker associated with progressive disease, we did not observe an increase in C1q prior to IGRA conversion, neither in serum nor in BAL (Fig. 4i,j). Not earlier than two weeks post-IGRA conversion local C1q levels were elevated over baseline. This observation further asserts the association of C1q with progressive, rather than latent or incipient, tuberculosis disease. Finally, to further assert the relation between serum C1q and disease progression, we pooled the data of all studies described and correlated serum C1q levels with the amount of “local” pathology in the lung and with the amount of disseminated disease, i.e. lung draining lymph node pathology and extra-thoracic pathology. As expected, serum C1q levels determined at end-point correlated most strongly with disseminated disease (for lung draining lymph node pathology: Spearman’s rho = 0.697, P = < 0.0001, for extra thoracic pathology: Spearman’s rho = 0.635, P = < 0.0001). Correlation of serum C1q with the amount of lung pathology was significant, but weak (Spearman’s rho = 0.311, P = < 0.0001).

### Pulmonary BCG vaccination increases local C1q production

**Figure 5:**
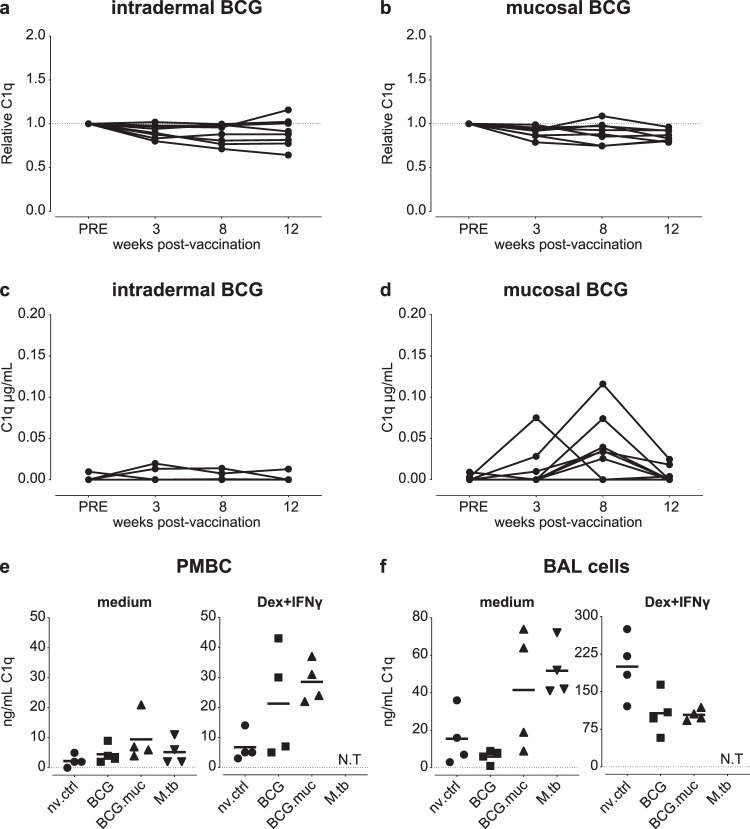
Pulmonary but not intradermal BCG vaccination results in upregulation of local production of C1q in rhesus macaques. (**a–d**) C1q levels in serum (**a,b**) and broncho-alveolar lavage (BAL) fluid (**c,d**) after BCG vaccination of rhesus macaques either through intradermal injection (BCG, **a**,**c**) or endobronchial instillation (BCG.muc, **b**,**d**). C1q production after stimulation with culture medium or dexamethasone plus interferon-γ (Dex + IFNγ) of (**e**) PBMC and (**f**) BAL cells, comparing from left to right non-vaccinated (nv.ctrl), standard BCG vaccinated, BCG.muc vaccinated, and low dose (15 CFU) *Mtb* infected rhesus macaques. N.T = not tested. Horizontal lines in (**e**,**f**) indicate group medians.

C1q is considered to contribute to control of infection by enhancing phagocytosis of pathogens, either by opsonization and specific uptake or by activating phagocytes through engagement of C1q-binding receptors^33^. As we have previously found mucosal BCG to be superior over intradermal BCG in preventing TB infection and disease, we here investigated BCG’s capacity to induce upregulation of C1q. We determined C1q in serum and BAL fluid from intradermal and mucosal BCG vaccinated animals from the repeated low dose challenge study described above and in Fig. 4. Similar to what has been described for humans, BCG vaccination in rhesus macaques by either route of administration, does not lead to upregulation of serum C1q (Fig. 5a,b). Interestingly, pulmonary, but not intradermal, BCG vaccination resulted in a marked increase in local C1q either 3 or 8 weeks after vaccination, to return to baseline levels by week 12 after vaccination (Fig. 5c,d).

As we observed a local, but not peripheral, elevation of C1q levels, we hypothesized that local production of C1q could underlie the increase observed in the mucosally vaccinated animals. We sought to verify the possibility of local C1q production by *in vitro* stimulation of BAL cells versus Peripheral Blood Mononuclear Cells (PBMCs) from BCG vaccinated rhesus macaques (intradermal as well as mucosal, at 8 weeks after vaccination), and from low dose *Mtb-*infected animals (at 11 weeks after infection) and untreated controls. Cells were incubated either with culture medium to assess *ex vivo* C1q production or with dexamethasone plus IFNγ (Dex/IFNγ), a positive control stimulus known to induce C1q release^17^, to assess the potency of these cells to produce C1q.

In PBMCs, a trend towards higher production by unstimulated PBMCs from vaccinated or *Mtb* infected animals (Fig. 5e, left panel) was observed when compared to unvaccinated/uninfected control animals. Prior BCG vaccination also seemed to potentiate C1q secretion of PBMCs, as reflected by the increase in C1q production in response to Dex/IFNγ stimulation in the vaccinees (Fig. 5e, right panel). Locally, *ex vivo* C1q production was highest in unstimulated BAL cells of animals that were exposed to either mucosal BCG or *Mtb* (Fig. 5f, left panel). After Dex/IFNγ stimulation, lower C1q production was observed in BCG vaccinated animals compared to non-vaccinated controls, potentially reflecting alterations in the cellular composition of the BAL (Fig. 5f, right panel)^26^. Collectively, our data show that pulmonary mycobacterial exposure can induce the production of local C1q, likely by resident alveolar macrophages.

## Discussion

In this work, we show that increasing C1q levels in serum and BAL are associated with TB disease severity in various non-human primate TB studies. High C1q levels did not precede detection of *Mtb* infection by IGRA conversion, but did correlate with TB pathology after both high (500 CFU) and low (<10 CFU) dose *Mtb* challenge in different macaque species. Likewise, in a long-term follow up (up to 1 year) setting, we found increasing C1q levels to be associated with reduced survival. Contrarily to what has been described for serum C1q, increasing pulmonary C1q was not exclusive to infection with *Mtb*; pulmonary vaccination with BCG also resulted in a temporal increase of C1q in BAL fluid. Whether this local C1q production plays a role in protection against *Mtb* infection and disease remains to be investigated.

Our data demonstrates that, in addition to being a marker of active TB in humans, C1q can also serve as a marker of progressive disease in experimental *Mtb* infection studies in NHP. C1q levels measured after *Mtb* infection correlated with disease severity measured post-mortem, but also with disease severity measured during infection by PET-CT. As C1q can be readily measured over the course of *Mtb* infection, it could therefore be applied to monitor TB disease progression in a resource-limited setting. Especially in cynomolgus macaques, which are known to develop latent TB infection^24^, measuring C1q at regular intervals after *Mtb* infection would be informative when assessing the occurrence and reactivation of LTBI. While the cohort of cynomolgus macaques measured in this study was not followed long enough to confirm the establishment of such a latent infection, the lack of C1q upregulation in the majority of this group might be indicative of TB latency development in these animals. As no post-infection increase in serum C1q could be observed after protective BCG vaccination, C1q can be used to assess efficacy along the infection phase when evaluating new drug or vaccine regimens in the NHP model.

Next to being a marker of progressive disease, it is tempting to speculate that C1q might also play a role in shaping the protective immune response observed after mucosal vaccination with BCG. Macrophages, including alveolar macrophages, express receptors, such as gC1qR and cC1qR (calreticulin), that can interact with either the globular head or collagen-like tail of C1q^11^. Not only does binding of (pathogen-bound) C1q to these receptors improve pathogen uptake^33^, C1q-receptor interaction can also modulate macrophage inflammatory status and cytokine responses. Monocytes incubated with C1q have been described to produce higher amounts of anti-inflammatory IL-10^34^, and it has been shown that C1q can polarize macrophages to an anti-inflammatory M2 phenotype, associated with the resolving of inflammation and tissue repair^35^. After mucosal BCG vaccination we observed an increase in pulmonary C1q (Fig. 5b), which coincides with increased PPD-specific IL-10 production by BAL cells^26^. This increased IL-10 production in response to PPD is sustained up to several weeks after *Mtb* challenge, and might be required to counterbalance the inflammatory response induced by BCG/*Mtb*, to prevent inflammation-induced damage^36^. However, both IL10 production and M2 polarization have also been identified as detrimental in the context of *Mtb* infection^37,38^, so the exact contribution of C1q to TB disease development remains to be elucidated. Lastly, C1q has the capacity to reduce production of IFNα^39,40^, which has been implied as detrimental in the (late phase) host immune response to *Mtb*^41,42^. In addition to modulating innate immune responses, C1q has also been described in the regulation of adaptive immune responses, either indirectly through regulating APC function or through direct interaction with C1q-receptors expressed on T-cells. However, reports on the capacity of C1q to directly activate T-cells are contradictory and need further investigation^43,44^. To fully appreciate the mechanistic role of C1q in shaping the protective immune response after mucosal BCG vaccination further research is required.

In summary, this work corroborates and extends on findings in human TB patients and links increased C1q levels with TB disease severity. It demonstrates that macaques can serve as informative model animals to study the role of (pulmonary) C1q in TB pathogenesis and/or protective immunity.

## Materials and Methods

### Animals & Ethics

All housing and animal care procedures took place at the Biomedical Primate Research Centre (BPRC) in Rijswijk, the Netherlands. The BPRC is accredited by the American Association for Accreditation of Laboratory Animal Care (AAALAC) and is compliant with European directive 2010/63/EU as well as the “Standard for Humane Care and Use of Laboratory Animals by Foreign Institutions” provided by the Department of Health and Human Services of the US National Institutes of Health (NIH, identification number A5539-01). Before the start of each study ethical approval was obtained from the independent animal ethics committee (in Dutch: Dierexperimentencommissie, DEC), as well as BPRC’s institutional animal welfare body (in Dutch: Instantie voor Dierwelzijn, IvD).

Animals included in each study were screened negative for prior exposure to mycobacteria by means of tuberculin skin testing with Old Tuberculin (Synbiotics Corporation, San Diego, CA) and an IFNγ ELISPOT against Purified Protein Derivative (PPD) from *Mycobacterium bovis*, *Mycobacterium avium* (both Fisher Scientific, USA) or *Mycobacterium tuberculosis* (Statens Serum Institute, Copenhagen, Denmark).

For the duration of the study animals were socially housed (pair-wise) at animal biosafety level 3. Animal welfare was monitored daily. Macaques were provided with enrichment in the form of food and non-food items on a daily basis. Animal weight was recorded prior to each blood collection event. To limit possible discomfort due to severe TB disease humane endpoints were predefined. All animal handling and biosampling was performed under ketamine sedation (10 mg/kg, by intra-muscular injection). When performing endobronchial challenge with *Mtb* or BAL, ketamine sedation was supplemented with intramuscular medetomidine (0.04 mg/kg) and an analgesic sprayed into the larynx. At the end of the study or when reaching a humane endpoint, animals were euthanized by intravenous injection of pentobarbital (200 mg/kg) under ketamine sedation. Veterinary staff and animal care-takers were blinded to animal treatment.

### *Mtb* challenge

In all studies, animals were challenged with *Mycobacterium tuberculosis* Erdman K01 strain (BEI Resource, VA, USA). Every *Mtb* challenge occurred by endobronchial instillation, targeting the lower left lung lobe. All challenge events were executed in a single session within 2–3 hours from preparing the inoculum from frozen *Mtb* stock, challenging animals in random order. *Mtb* challenge dose, verified in each study by quality control plating, is indicated in the relevant results sections and figure legends.

### Serum and BAL-fluid collection

Serum and BAL fluid were collected at various timepoints after *Mtb* challenge or BCG vaccination, as indicated in the figure axes. For serum, peripheral blood was collected in serum separator tubes by means of venipuncture. Tubes were spun for 10 minutes at 1000 g to harvest cell-free serum, which was stored at −80 °C pending further analysis. BAL was performed by targeting the lower left lung lobe by bronchoscope, followed by instillation and recovery of three times 20 mL prewarmed 0.9% saline solution. BALs were first passed over a 100 μm filter to remove mucus and debris, followed by centrifugation for 10 minutes at 400 g. Supernatant was decanted and stored at −80 °C pending further analysis. Serum and BAL fluid were filter-sterilized by centrifugation through 0.2 μm PVDF membrane plates (Fisher Scientific) before analysis.

### Post-mortem pathology scoring

Post-mortem tuberculosis pathology was scored by a semi-quantitative grading system (adapted from^45^) based on lesion size, manifestation and frequency, and lymph node involvement. Over time, from one to the other study, the scoring system has been adjusted slightly on minor details to more accurately describe disease manifestation, but the overall algorithm, as outlined in the paragraph below, has remained the same. When comparing disease severity between studies, pathology scores have been expressed as a percentage of the maximal possible score.

After euthanasia, the thoracic cavity, including the heart, ribcage, vertebrae and diaphragm were all macroscopically scored for the presence of granulomas and pleural adhesions. Lungs were isolated and lobes were separated from the trachea. Subsequently, lung lobes were cut in 5 mm thick slices and scored for the amount of pathology. Lung draining lymph nodes were removed from the trachea and scored for size and extent of involvement. Extra-thoracic organs such as kidneys, spleen, pancreas and liver were macroscopically assessed for the presence of lesions. The “Total Pathology score” that is depicted throughout represents the summed score of all these organs.

### C1q production by PBMC and BAL cells

To assess production of C1q by PBMC and BAL cells, freshly isolated cells were taken up in Roswell Park Memorial Institution 1640 medium (RPMI), supplemented with 10% Fetal Calf Serum (FCS), glutamine and penicillin/streptomycin. Cells were seeded at 200.000 cells per well in triplicate in 96-well round bottom plates and stimulated with 4 μg/mL dexamethasone (Merck) +200 U/mL IFNγ (Peprotech, UK), or left untreated. Supernatants were harvested after 96 hours, pooled and filter-sterilized by centrifugation through 0.2 μm PVDF membrane plates (Fisher Scientific) before storage at −80 °C and subsequent analysis by ELISA.

### C1q ELISA

C1q levels in sera, BAL fluid and culture supernatants were measured using an in-house developed ELISA, as described previously^18^. In brief, 96-well Maxisorp plates (Nunc) were coated overnight with mouse anti-human C1q (Nephrology department, LUMC) in coating buffer (0.1 M Na_2_CO_3_, 0.1 M NaHCO_3_, pH9.6). The next day, plates were washed with PBS/0.05% Tween and blocked with PBS/1%BSA. After subsequent washing, samples (serum diluted 1:4000, BAL fluid 1:1, culture supernatant 1:1) were added to the plates. A serially diluted pool of normal human serum (NHS) was taken along as a standard. Bound C1q was detected by incubation with rabbit anti-human C1q, followed by goat anti-rabbit HRP (both Dako) and ABTS substrate. C1q concentration is calculated in μg/mL from a human C1q standard.

### Statistics

Statistical analyses were performed using Graphpad Prism, software version 7. Significance of differences between groups was calculated by two-sided Mann-Whitney testing. All correlations were calculated by Spearman’s log rank testing.

## Supplementary information

Supplementary information

## Data Availability

The datasets generated and analyzed are available from the corresponding authors upon reasonable request. Likewise, biomaterials remaining from the studies described could be shared for further research.
