# Supplementary material for: Systemic and pulmonary C1q as biomarker of progressive disease in experimental non-human primate tuberculosis
Source: Sci Rep. 2020 Apr 14;10:6290. doi: 10.1038/s41598-020-63041-4 (PMC7156429; doi:10.1038/s41598-020-63041-4)
Supplement: Supplementary file 1 — Supplementary information [file 41598_2020_63041_MOESM1_ESM.pdf]

Systemic and pulmonary C1q as biomarker of progressive disease in experimental non-human primate tuberculosis

Karin Dijkman[1], Rosalie Lubbers[2], Nicole V. Borggreven[3], Tom H.M. Ottenhoff[4], Simone A. Joosten[4], Leendert A. Trouw[3], Frank A.W. Verreck[1*].*

[1] from the section of TB Research & Immunology, department of Parasitology, Biomedical Primate Research Centre (BPRC), Rijswijk the Netherlands, and

the departments of [2] Rheumatology, [3] Immunohematology and Blood Transfusion, and [4] Infectious Diseases, Leiden University Medical Centre (LUMC), Leiden, the Netherlands

Supplemental Figure 1

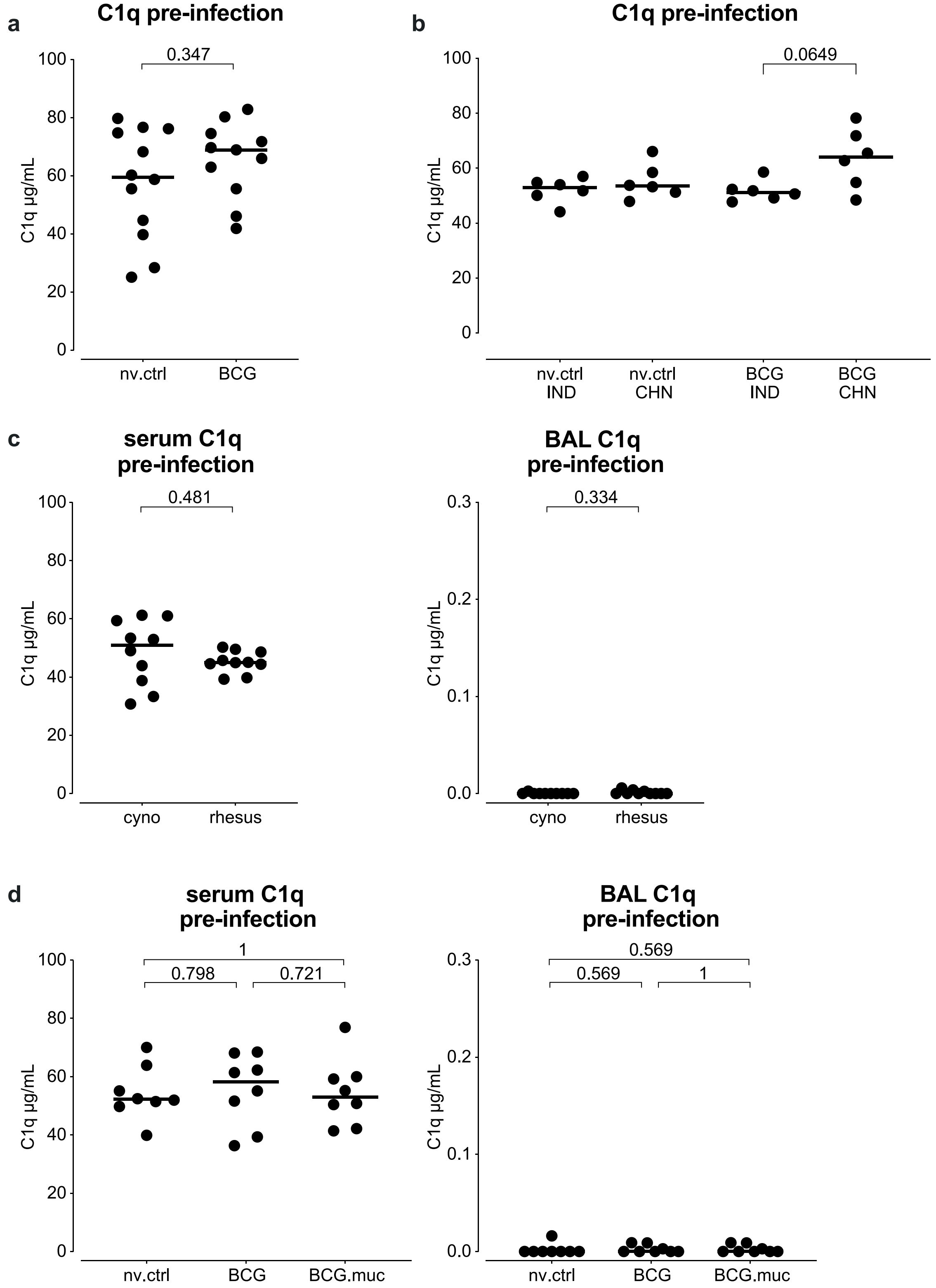

Supplemental figure 1. Pre-infection C1q levels for each study

C1q levels before infection with *Mtb* for each of the cohorts described in the main body text. **a)** Pre-infection serum C1q levels per treatment group from the high-dose *Mtb* challenge with long (1 year) follow-up described in **Figure 1**. **b)** Idem from the high-dose *Mtb* challenge study with short (3 months) follow-up described in **Figure 2**. **c**) Pre-infection serum (left panel) and BAL C1q levels (right panel) from the low dose *Mtb* challenge study described in **Figure 3**. **d**) Idem from the repeated limiting dose *Mtb* challenge study described in **Figure 4**.

Horizontal lines in **a-d** indicate group medians. Statistical significance of group differences determined by two sided Mann-Whitney.

Supplemental Figure 2

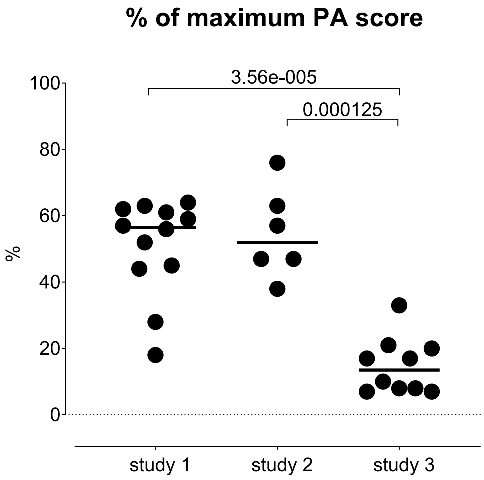

Supplemental figure 2. Comparison of TB associated pathology after high and low dose *Mtb* challenge.

Pathology (expressed as percentage of the maximal possible score) measured at endpoint after high dose *Mtb* challenge (500 CFU), long-term follow up (study 1), high dose, short-term follow up (study 2) and low dose (<10 CFU), short-term follow up (study 3).

Horizontal lines indicate group medians. Statistical significance of group differences determined by two sided Mann-Whitney.
